# Preliminary evidence that daily light exposure enhances the antibody response to influenza vaccination in patients with dementia

**DOI:** 10.1016/j.bbih.2022.100515

**Published:** 2022-09-20

**Authors:** Mirjam Münch, Rolf Goldbach, Naomi Zumstein, Petra Vonmoos, Jean-Louis Scartezzini, Anna Wirz-Justice, Christian Cajochen

**Affiliations:** a Solar Energy and Building Physics Laboratory, Environmental and Civil Engineering Institute, Ecole Polytechnique Fédérale de Lausanne, Lausanne, Switzerland; b Centre for Chronobiology, Psychiatric Hospital of the University of Basel, Basel, Switzerland; c Transfaculty Research Platform, Molecular and Cognitive Neurosciences, University of Basel, Basel, Switzerland; d Geriatric Service of the City of Zurich, Zurich, Switzerland; e Department of Anthropology, McGill University, Montreal, Canada; f University Research Priority Program “Dynamics of Healthy Aging”, University of Zurich, Zurich, Switzerland; g Sonnweid – the Home, Wetzikon, Switzerland

**Keywords:** Immunology, Flu shot, Circadian, Light, Neurodegeneration, Rest-activity, Sleep, IS, Inter-daily Stability, IV, Intradaily Variability, SCN, Suprachiasmatic Nucleus

## Abstract

Enhancing lighting conditions in institutions for individuals with dementia improves their sleep, circadian rhythms and well-being. Here, we report first findings that exposure to brighter light during daytime may support the immune response to the annual influenza vaccination. Eighty older institutionalised patients suffering from dementia (54 women and 26 men) continuously wore an activity tracker for 8 weeks to assess individual light exposure and rest-activity cycles. We analysed the patients’ immune response from two blood samples taken before and 4 weeks after the annual influenza vaccination. Individual antibody concentrations to three influenza virus strains (H3N2, H1N1, IB) were quantified via hemagglutination inhibition assays. By quantifying individual light exposure profiles (including daylight), we classified the patients into a low and a high light exposure group based on a median illuminance of 392.6 lux. The two light exposure groups did not differ in cognitive impairment severity, age or gender distribution. However, patients in the high light exposure group showed a significantly greater circadian rest-activity amplitude (i.e., more daytime activity and less nighttime activity) along with a significantly greater antibody titer increase to the H3N2 vaccine than patients in the low light exposure group, despite similar pre-vaccination concentrations. Sufficient seroprotective responses to all three influenza virus strains were attained for ≥75% of participants. These data provide preliminary evidence for a potentially enhanced immune response in patients with dementia when they received more daily light. Future studies are needed to determine whether regular daily light exposure may have beneficial effects on the human immune system, either directly or via a stabilising circadian sleep-wake rhythms.

## Highlights

• Data from individual light exposures and circadian rest-activity cycles in 80 institutionalised patients with dementia.
• Blood samples were taken before and 4 weeks after the annual influenza vaccination.
• Patients with on average higher light exposure had higher specific antibody titer ratios after the influenza strain H3N2 vaccine.
• Circadian rest-activity amplitude and inter-daily stability were also higher in the group with higher daily light exposures.
• The results provide preliminary evidencefor beneficial effects of light on the immune system in patients with dementia.

## 1 Introduction

In older patients with dementia, the decline of cognitive functions is often accompanied by disturbances in sleep-wake rhythms as well as alterations in mood, behaviour and daily activities (Wennberg et al., 2017; Lee and Lyketsos, 2003). In this context, it is well documented that the neurons of the central circadian pacemaker in the brain, the suprachiasmatic nucleus (SCN), undergoes a progressive decline with dementia (Swaab et al., 2002), resulting in a decrease in circadian rhythm amplitude and fluctuations in circadian phase with consequently weaker entrainment to the 24-h day (Musiek et al., 2018). These changes in the SCN, together with the natural age-related alterations in the visual system, contribute to deterioration in mood, sleep-wake cycles and behaviour, and together, these symptoms increase the need for further care and medication prescriptions (Van Erum et al., 2018).

Environmental light exposure is the strongest Zeitgeber, (defined as an environmental signal that entrains biological rhythms in organisms; it was coined from the German word ‘time giver’) to synchronise the biological clock in the SCN with the solar 24-h light dark cycle (Aschoff, 1965). Therefore, bright light exposure can increase Zeitgeber strength on various functions of the circadian system in humans. Clinically, exposure to bright light at the appropriate time of day improves behavioural symptoms as well as circadian sleep-wake cycles in older demented patients (Roccaro et al., 2020; Ancoli-Israel et al., 2003; Wahnschaffe et al., 2017; Mishima et al., 1998; Figueiro et al., 2019; Münch et al., 2017; Riemersma-van der Lek et al., 2008; Van Someren et al., 1997; Sloane et al., 2007) and also slows cognitive deterioration down (Riemersma-van der Lek et al., 2008) (for a systematic review see (Hjetland et al., 2020)). Simulated dawn and dusk at the bedside of institutionalised demented patients was found to advance nocturnal sleep onset by 1 h (Fontana Gasio et al., 2003) and improved morning mood and well-being (Bromundt et al., 2019). Other factors associated with cognitive decline and aging contribute to the weakening of circadian rhythms, including functional limitations interfering with physical activity. These could impede exposure to Zeitgebers and deteriorate circadian rhythms.

Recurrent influenza epidemics accelerate mortality among institutionalised older patients (Thompson et al., 2003) and are one of the 10 leading causes for deaths in the USA. The mortality risk can be significantly decreased by winter flu shots (Weinberger et al., 2008), which are recommended for older patients in long-term care institutions (Goodwin et al., 2006). However, influenza vaccination is less effective in older than young individuals (Frasca and Blomberg, 2014), and the immune response is attenuated with age (Weinberger et al., 2008; Henry et al., 2019). IgA and IgG antibody concentrations decrease with age, with a faster decline of the antibody titers (Goodwin et al., 2006), especially in very old and frail adults. Consequently, older individuals are likely to be insufficiently protected by vaccination (Grubeck-Loebenstein and Wick, 2002; Monto et al., 2009). More recently, during the COVID-19 pandemic, it was shown that the older population, especially those living in institutions (such as nursing homes) were most vulnerable to infections (Kim and Su, 2020; Chidambaram, 2020; Blanco-Tarrio and Blanco Sánchez, 2020), along with a significantly higher mortality risk when positively tested for COVID-19 (Fisman et al., 2020).

There is also a (bidirectional) role for sleep (Besedovsky et al., 2019; Irwin and Opp, 2017) and the circadian clock in different immune functions (Haspel et al., 2020; Born et al., 1997; Cermakian et al., 2013), where the SCN essentially modulates innate and adaptive immune responses (reviewed in (Scheiermann et al., 2018; Labrecque and Cermakian, 2015; Cermakian et al., 2022)). Aberrant light exposure (such as with shift work or jet lag) can induce phase shifts in many circadian clock-controlled functions, including immune responses (Hayashi and Kikuchi, 1985; Abele et al., 2019). The consequences of such desynchronisation are dampened circadian amplitudes of SCN cell expression in animals (Inokawa et al., 2020). In humans, misaligned circadian rhythms appear to be linked to severe health problems such as a higher risk for cardiovascular, metabolic and neurodegenerative disease, cancer and impaired immune function (Haspel et al., 2020). Thus, decreased circadian amplitude is likely to impair the ability to respond to infections. So far, only a few studies have addressed the direct impact of ocular light exposure on the human immune system (Roberts, 2000). One study showed that exposure to continuous bright light during daytime (i.e., polychromatic white electric light; 5000 lx; between 6:30am and 10:30pm) significantly increased unspecific salivary IgA antibody formation in healthy young subjects (Park and Tokura, 1999). The authors concluded that brighter light exposure during daytime activated a greater immune response in human mucosa cells (Park and Tokura, 1999).

Enhanced daytime light exposure can improve sleep (Figueiro et al., 2014) and increases circadian amplitude of rest-activity cycles in older, institutionalised individuals (including those suffering from dementia) (Ancoli-Israel et al., 2003; Van Someren et al., 1997). It is unknown whether daily exposure to bright light might also ameliorate adaptive immune responses indirectly, by increasing the strength of external Zeitgebers to stabilise the circadian system, or directly via an acute action on brain areas regulating immune responses. One example of such an adaptive immune reaction is the production of specific antibodies in response to an influenza virus vaccination.

Thus, this study aimed to assess vaccine responses to three different virus strains of the annual vaccine in a cohort of institutionalised patients with severe dementia. We hypothesised that patients with higher daily light exposure over several weeks would show increased antibody titers in response to the influenza vaccination than patients with lower daily exposure.

## 2 Methods

### 2.1 Study design

The cross-sectional study occurred for 8 weeks in fall/winter in a nursing home in Wetzikon (Zurich, Switzerland) in 2012. Patients in twelve different wards spent time in dayrooms equipped with conventional or ‘dynamic lighting’ systems, where illuminance and correlated colour temperature varied across the day (Münch et al., 2017). We have previously reported the results from patients' daily activities, agitation, alertness mood, quality of life (from questionnaires assessed by staff members), rest-activity cycles, sleep (derived from activity monitors), and melatonin concentrations (from saliva samples) (Münch et al., 2017). Here we present the results of the specific antibody responses to the annual influenza vaccination.

### 2.2 Participants

The study group comprised patients over 50 years with one of the following dementia diagnoses (according to DSM-IV): vascular dementia, Alzheimer dementia, frontotemporal dementia, Parkinson's dementia or mixed forms of dementia. Initially, 104 patients were included in the study (Münch et al., 2017). Here, only patients who had worn the activity watches, received the annual flu shot, and had given two blood samples, were included in the analysis (n = 80; see Table 1 for demographics and main comorbidities, and Supplemental Table S1 for exclusion details). None of the patients was visually blind. The mean age was 78.3 ± 8.9 years (54 women, 26 men; range 55–95 years). Ethical approval for all study procedures was obtained from the local Ethical Review Board (KEK, Zurich, Switzerland, protocol # KEK-ZH, 2012–0059, now SWISSMEDIC). In addition, written informed consent for study procedures was obtained from family members or legal representatives prior to study start.

**Table 1:** Demographics and comorbidities (derived from medical charts): AD = Alzheimer Dementia; MT = Mixed Dementia; P=Parkinson Dementia; U=Unspecified dementia type; V=Vascular; S-MMSE = Severe-Mini-Mental Status Examination. Comorbidities are listed according to the medical diagnosis (medical chart). TIA = transitory ischemic attack; PRIND = prolonged reversible ischemic neurological Deficit;^a^ = difference between both light groups is not statistically significant (p > 0.05).

Demographics:	Low Light Group (n = 40;	High light group (n = 40)
Age (mean ± SD)^a^	79.0 ± 9.2	77.7 ± 9.2
Sex	27 w/13 m	27 w/13 m
Type of Dementia	AD 21; FT 1 MT 11; P 1; U: 6; V 0	AD 16; FT 2; MT 9; P 1; U 11; V 1
S-MMSE (mean ± SD)^a^	8.1 ± 9.8	7.8 ± 9.6

**Comorbidities (occurrence, N)**		
Cancer	5	5
Cardio-vascular (Hypertension, Congenital heart failure, coronary artery disease)	24	17
Chronic respiratory disease (asthma, chronic obstructive pulmonary disease, sleep apnea)	3	2
Kidney disease	6	5
Liver/Pancreas disease	1	
Metabolic disease (Diabetes mellitus, obesity)	3	5
Gastrointestinal (reflux oesophagitis, stomach ulcera, colitis)	7	3
Epilepsy	4	9
Brain Insult (TIA, PRIND, ischemic attacks)	3	3
Depression	21	20

### 2.3 Individual light exposures

Individual light exposure (illuminance) was recorded via wrist-worn activity monitors equipped with a calibrated light sensor (Motion Watch 8 ®, Camntech, UK). All recordings were downloaded weekly to a PC and visually inspected. Trained assistants visually inspected and edited light and rest-activity data (see (Münch et al., 2017) for a detailed description). In brief, if there was a 24-h day with a gap of recorded rest-activity lasting less than 3 h, this gap was edited with the 24-h mean of this person. If there was a gap with no rest-activity data for more than 3 h, that 24-h period was excluded from further analyses. We also applied the same criteria for the light recordings except that if a 24-h period contained rest-activity, but no light data for more than 3 h (due to coverage of the sensor by sleeves), only the light data was not used, and if the gap was less than 3 h, the light data was also interpolated with the 24-h mean of that day.

Based on the averaged individual light exposure, two subgroups were created retrospectively, as assessed from wrist-worn light sensors (Motion Watch 8, Camntech, UK), similarly to what was done for the previously reported data (Münch et al., 2017). For this purpose, median illuminance across 8 weeks between 8:00 and 18:00 was calculated for each participant resulting in 426.1 ± 304.4 lux (mean ± SD). In a next step, a median split of these data (n = 80) resulted in a group with higher mean light exposure (= high light group, i.e., >392.65 lx; n = 40, 27 women, 13 men) and a group with lower mean light exposure (= low light group, i.e., <392.65 lx; n = 40, 27 women, 13 men). The two light exposure groups did not differ in age (low light group = 79.0 ± 9.2 years; high light group = 77.7 ± 8.6 years; p = 0.6; Wilcoxon 2-sample test), or cognitive impairment, as assessed by the Severe-Mini Mental State Examination (S-MMSE before the flu shot (Harrell et al., 2000)). The S-MMSE score was 8.1 ± 9.8 for the low light group and 7.8 ± 9.6 for the high light group (p = 0.93 Wilcoxon 2-sample test). Both groups also showed comparable comorbidities (see Table 1).

### 2.4 Rest-activity cycles and sleep

From the wrist-worn monitors, circadian rest-activity data were derived as described in (Münch et al., 2017) and above. Bed and wake times were assessed visually by scoring each 24-h rest-activity recording by a trained assistant. In brief, a 24-h recording entered statistical analysis if there was less than 3 h of missing data. In addition, habitual bed- and waketimes, communicated by carers were additionally used to support decisions. Especially since there were patients, who went to bed at ‘their’ habitual time – but then got up again, wandered around and went back to bed and so forth, showing a typical pattern of sleep-wake fragmentation and sometimes night time agitation. Two scores independently re-scored these recordings for difficult cases until consent was achieved. All data, which included 24-h days per patient underwent a non-linear circadian regression analysis (Van Someren et al., 1997, 1999), resulting in the following variables: inter-daily stability (IS), inter-daily variability (IV), and relative amplitude (RA). The RA is defined as the ratio of the 10 h with the highest activity (M10) relative to the 5 h with the lowest activity (L5) per 24 h. Sleep variables from night time sleep episodes were determined by the software Sleep Analysis v7.23 (Camntech UK). Bed- and wake times were assessed as described in (Münch et al., 2017) (summarised above) and sleep variables were re-analysed for the 80 participants: habitual bedtime, wake time, time in bed, wakefulness during scheduled sleep, sleep duration, sleep efficiency (ratio between sleep duration: time in bed) and sleep fragmentation index.

### 2.5 Blood samples and influenza vaccination

Two blood samples were obtained by standard venous puncture, one immediately before and one approximately 4 weeks after the annual influenza vaccination. A total of 8 ml of blood was drawn from each patient by professional staff members of the nursing home before noon. The blood sample was sent to an external laboratory for general analyses (Medica AG, Zürich, Switzerland). For the specific antibody titer analysis (Prof. A-C Siegrist, University of Geneva), 4 ml of whole blood was coagulated within 1 h after the sample was taken at room temperature, centrifuged. Then, the serum was pipetted into tubes (Eppendorf ®) and immediately frozen at −20 °C before sending to the University of Geneva for hemagglutination inhibition assays (HIA) (Gabay et al., 2011).

The vaccination was performed in week 44 (i.e., between Oct. 29th and Nov. 4th, 2012; according to national recommendations in Switzerland), except for three patients who received the first blood sample and the flu shot in week 46 and the second blood sample at the end of week 50 (due to an acute infection in week 44). The trivalent vaccine Fluarix ® (GlaxoSmithKline Biologicals, UK) was applied via intramuscular injection in the patients' upper arm by the nursing home staff. The vaccine contained attenuated virus particles against influenza A virus (H3N2, H1N1) as well as against influenza B virus (IB). The strains matched the World Health Organization (WHO) recommendations for the influenza season 2012/2013. Immune responses were calculated only in patients who received the flu shot and had no acute infection (i.e., less than 15′000 Leucocytes/μl), which resulted in 80 patients. Patients who had very high antibody titer concentrations (>1000) in the pre-vaccination blood sample for one of the three virus strains were also excluded from the analysis of that virus strain, which was the case in two patients for the H3N2 antibody titer, and in one patient for the H1N1 antibody titer.

### 2.6 Statistical analysis

For rest-activity cycles and sleep, the same analyses as described in (Münch et al., 2017) were performed for the subset of participants undergoing the antibody titer analysis (n = 80). For influenza vaccination, the pre- and post-vaccination antibody titers and the ratio (pre-vaccination/post-vaccination) were used to compare the two light exposure groups. The fixed factors LIGHT EXPOSURE GROUP (low light vs. high light), AGE as a categorical factor (on dichotomized variables derived from median split of age, i.e., <80.0 or >79 y), and SEX as well as their interactions were added to the model. Subject was added as a random factor. For general blood variables, the repeated factor SESSION (i.e., pre- and post-vaccination blood sample) was included. The factor COGNITIVE IMPAIRMENT (i.e., individual S-MMSE scores) was also added, and results were reported separately to test whether the degree of cognitive impairment was a confounder. Statistical comparisons were performed with a generalized linear mixed model (GLIMMX; with a lognormal distribution if the data was not normally distributed) by using the Software Package SAS (SAS Institute Inc., Cary, NC, USA; v 9.4). Degrees of freedom were determined with the Satterthwaite approximation, and post-hoc comparisons were performed with the Tukey-Kramer test (corrected for multiple comparisons).

In order to compare the overall immune protection against the influenza virus, we determined geometric mean titers (GMTs) for pre- and post-vaccination values (Gabay et al., 2011). The differences between high and low light exposure groups for post-vaccination GMTs were compared by survival analyses (Kaplan-Meier on log-ranked values; Sigma Plot v11.0, Statsoft Software Inc). For both light exposure groups, seroprotection rates (which are defined as percentage of patients with post-vaccination antibody titer ≥1:40) and seroconversion rates (described in reference (Gabay et al., 2011)), defined as percentage of patients with 4-fold increase of pre-vaccination GMT titer, were also calculated.

## 3 Results

### 3.1 Rest-activity cycles and sleep

Rest-activity cycles revealed a significantly higher inter-daily stability (IS) and relative amplitude (RA) in the high light group than the low light group (Table 2; main effect of LIGHT EXPOSURE GROUP; n = 80; F_1,72_ > 4.9, p < 0.03). In general, IS and RA were higher in women than men (IS: women: 0.39 ± 0.14, men: 0.30 ± 0.14; means, SD; RA: women: 0.73 ± 0.15, men: 0.62 ± 0.18; main effect of SEX; F_1,72_ > 6.4 p < 0.02). Activity during the 5 h with lowest daily activity (L5) was significantly higher in men (861 ± 572, arbitrary units) than in women (594 ± 607 main effect of SEX; F_1,72_ = 4.5; p = 0.04), and men also had longer wakefulness during scheduled sleep than women (men: 2.1 h ± 1.1; women 1.8 h ± 1.2; main effect of SEX; F_1,72_ = 5.17; p = 0.03), which did not result in any other significant differences of sleep duration or time in bed between men and women. There were no significant main effects or interactions for the remaining circadian or sleep variables. In order to test whether the degree of functional impairment had an impact on the results, we added the scores from SSME to the regression model. The main differences for RA between both light groups remain the same, i.e., higher RA for participants in the high than the low light group (main effect of LIGHT EXPOSURE GROUP; F_1,50_ = 4.96; p = 0.03). For IS, the difference between both light groups changed from statistical significance to a statistical trend (p = 0.1) and sleep fragmentation became worse in men than in women, with lower SE and shorter sleep duration (main effect of SEX; F_1,50_ > 4.47; p < 0.04).

**Table 2:** Circadian and sleep variables (derived from activity monitors) for low and high light exposure (low LE, high LE) groups (mean, SD in brackets; n = 80). IS = Inter-daily stability; IV = intra-daily variability; L5 = 5 h with lowest activity; M10 = 10 h with highest activity; RA = relative amplitude (see Ref (Van Someren et al., 1999) for more details); BT = habitual bedtime (h); WT = habitual waketime (h); TIB = Time in bed (h); Wake = waketime during scheduled sleep (h); Sleep duration (h); SE = sleep efficiency (%; sleep time/TIB x 100); Fragmentation Index (dimensionless). * = p < 0.05 between light exposure groups (bold).

Variable	Low Light Exposure Group	SD	High Light Exposure Group	SD
IS *	**0.33**	**(0.15)**	**0.38**	**(0.14)**
IV	1.05	(0.30)	1.18	(0.38)
L5	705.77	(591.84)	655.15	(625.49)
M10	3969.53	(3263.18)	4241.26	(3756.49)
RA *	**0.66**	**(0.18)**	**0.72**	**(0.15)**
BT (h)	19.50	(1.00)	19.57	(1.31)
WT (h)	8.24	(0.71)	8.10	(0.79)
TIB (h)	12.71	(1.25)	12.52	(1.73)
Wake (h)	2.14	(1.26)	1.84	(1.09)
Sleep duration (h)	10.28	(2.26)	10.40	(2.48)
SE (%)	80.09	(12.08)	82.23	(10.90)
Fragmentation Index	50.81	(24.87)	46.69	(18.86)

### 3.2 Blood variables

There was no difference between the two light exposure groups for any blood variables in between the pre- and post-vaccination samples (p > 0.13, Table 3). In general, the counts of leucocytes and neutrophil lymphocytes and the percentage of large unstained cells (LUC) were higher in the pre-than the post-vaccination sample (main effect of SESSION; p < 0.05; Table 3), while the absolute lymphocyte count and percentage were higher in the post-than the pre-vaccination sample (p < 0.05). For the younger subgroup (see statistics) of patients, the erythrocyte counts (EC), haemoglobin (HG), haematocrit (HK), mean corpuscular haemoglobin concentrations (MCHC) and lymphocytes (in % from automated processing) were significantly higher than in the older patient group (main effect of AGE; p < 0.015).

**Table 3:** Blood analyses before and 6 weeks after the influenza vaccination. CRP = C-reactive protein, LC = leucocytes, EC = erythrocytes, HB = haemoglobin, HK = haematocrit, MCV = mean corpuscular volume, MCH = mean corpuscular haemoglobin, MCHC = mean corpuscular haemoglobin concentration, TC = thrombocytes, LUC = large unstained cells, FACS = fluorescence activated cell sorting (CD3, CD4, CD8); means and (SEM); n = 80; *= p < 0.05; main difference between pre- and post-vaccine session (bold).

Blood Marker	Pre-vaccination (SEM)		Post-vaccination (SEM)	
CD4 (count/μl)	879.23	(33.17)	892.61	(36.09)
CD8 (count/μl)	435.74	(35.63)	448.29	(33.43)
CD4/CD8 Ratio	2.76	(0.19)	2.65	(0.18)
**Lymphocytes (count/μl)** *	**1775.00**	**(86.17)**	**1881.01**	**(72.40)**
CRP (mg/l)	10.40	(2.03)	6.59	(1.03)
**LC (count/μl)** *	**7110.00**	**(243.31)**	**6602.50**	**(189.08)**
EC (G/l)	4.37	(0.06)	4.32	(0.05)
HB (g/l)	130.09	(1.70)	128.85	(1.57)
HK (l/l)	0.40	(0.005)	0.39	(0.004)
MCV (fl)	90.64	(0.52)	90.94	(0.48)
MCH (pg)	29.77	(0.19)	29.85	(0.16)
MCHC (g/l)	328.40	(0.87)	328.43	(0.78)
TC (G/l)	292.91	(8.60)	284.41	(8.24)
**Neutrophil (%)** *	**62.71**	**(1.17)**	**59.92**	**(1.13)**
Eosinophil (%)	3.66	(0.26)	4.12	(0.41)
Basophil (%)	0.53	(0.03)	0.54	(0.03)
Monocytes (%)	6.07	(0.19)	6.17	(0.15)
**Lymphocytes (%)** *	**23.94**	**(0.94)**	**26.50**	**(1.00)**
**LUC (%)** *	**3.08**	**(0.27)**	**2.76**	**(0.17)**
FACS (count/μl)	1331.20	(57.60)	1358.33	(55.55)

### 3.3 Influenza vaccination response

**Fig. 1:**
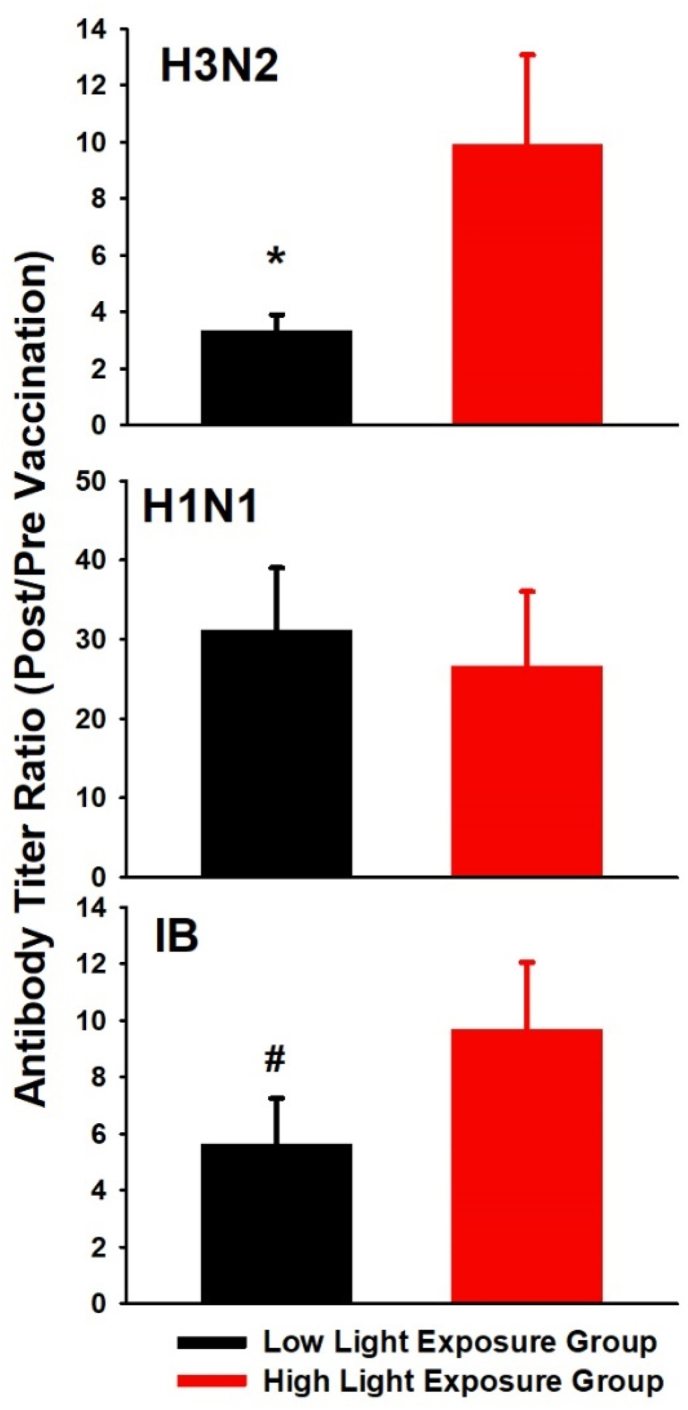
Mean values (+SEM) for antibody titer ratios (post-vaccination/pre-vaccination) for all three influenza strains and both sub-groups of patients [high (red bars) vs. low light exposure group (black bars)]: H3N2 (n = 78); H1N1 (n = 79); IB (n = 80). *: p = 0.01 (main effect of light exposure group) and trend: #: p = 0.08. . (For interpretation of the references to colour in this figure legend, the reader is referred to the Web version of this article.)

Pre- and post-vaccination antibody titers did not show significant differences between the light exposure groups (p > 0.07). However, the post/pre-vaccination ratios revealed a significantly higher increase in antibody titer for the H3N2 virus strain in the high than the low light group (Table 4; F_1,70_ = 6.8; p = 0.01; Fig. 1). On visual inspection, the IB post/pre-vaccination ratio seems similar to the H3N2, but the difference did not reach significance. It revealed a trend (p = 0.08) for slightly higher antibody titer in the high light exposure group (for significant effects with AGE and SEX see Supplemental Results). The inter-individual degree of cognitive impairment did not significantly modulate the differences in antibody titer ratios for the H3N2 virus strain between both groups, when adding the S-MMSE scores to the model (main effect of LIGHT EXPOSURE GROUP; F_1,48_ = 5.52; p = 0.02).

**Table 4:** Absolute influenza antibody titers pre- and post-vaccination for three virus strains: H3N2 (n = 78), H1N1 (n = 79) and IB (n = 80) as well as ratio (post/pre) for the low and high light group; means and (SEM). * = significant differences between ratio of the low and the high light exposure group (p < 0.05; main effect of light exposure group, in bold).

Titer	Low Light Exposure Group		High Light Exposure Group	
Pre (H3N2)	140.8	(12.3)	150.3	(25.3)
Post (H3N2)	421.3	(69.2)	627.5	(146.3)
Ratio (H3N2) *	**3.4**	**(0.6)**	**9.9**	**(3.2)**
Pre (H1N1)	39.5	(8.4)	62.5	(12.3)
Post (H1N1)	477.5	(93.8)	401.6	(112.6)
Ratio (H1N1)	31.1	(7.9)	26.7	(9.4)
Pre (IB)	65.4	(9.4)	56.0	(9.4)
Post (IB)	232.6	(61.2)	201.9	(29.5)
Ratio (IB)	5.6	(1.6)	9.7	(2.4)

**Fig. 2:**
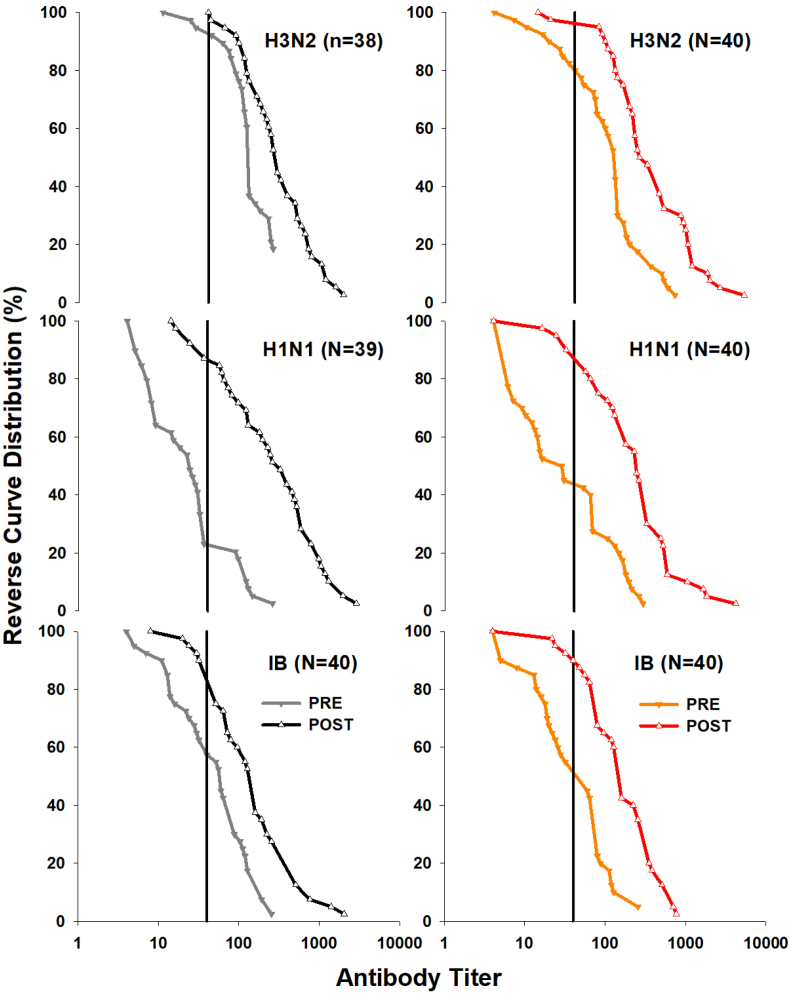
Reverse curve distribution plots from geometric mean antibody titers (GMT) for pre-vaccination and post-vaccination samples and the three influenza virus strains (H3N2, upper graph; H1N1, middle graph; IB, lower graph). The data is expressed in percentage for both light exposure groups of patients separately; left panel = low light exposure group; right panel = high light exposure group (filled grey and orange triangles down and grey lines and orange lines = pre-vaccination antibody titers; open black and red triangles up and black and red lines = post-vaccination titers). The vertical line in each graph represents the threshold for GMT titers of seroprotection by the influenza vaccination (i.e., a GMT ≥40). (For interpretation of the references to colour in this figure legend, the reader is referred to the Web version of this article.)

The geometric mean antibody titer (GMT) for pre- and post-vaccination responses was also calculated for both light exposure groups. From a cohort perspective, the post-vaccination seroprotection rates (i.e., the percentage of patients with a GMT greater or equal 40) were at least 75% (Supplemental Table S2). The seroconversion rates, which reflect a 4-fold increase of the GMT were greater or equal to 34% for all three virus strains. When performing a survival analysis on the reverse cumulative distributions of GMTs with the low and high light patient group (Fig. 2), there were no significant differences for the three influenza virus strains (Kaplan-Meier on log-ranked values; p > 0.4).

## 4 Discussion

A group of dementia patients with long-term brighter daytime light exposure showed significantly greater circadian inter-daily stability and higher relative amplitude of circadian rest-activity cycles along with higher antibody production in response to the influenza virus strain H3N2 than the patient group with lower bright light exposure. The seroprotection and seroconversion rates, which are standard criteria to evaluate the effectiveness of a vaccination within a cohort, showed that both light exposure groups were well protected against the influenza virus. However, in highly vulnerable patients, any additional benefit at the individual immunological response level is desirable and can improve general health in these vulnerable patients.

Even more so because the effects could have been indirectly conveyed via the improved circadian rest-activity cycles. Indeed, this assumption is corroborated by the significantly higher relative amplitude (RA) and greater inter-daily stability (IS) of rest-activity cycles in those participants with enhanced light exposure. A higher relative amplitude with greater inter-daily stability may have repercussions on other health related parameters, as shown for a variety of diseases (reviewed e.g., in (Abbott and Zee, 2019), (Leng et al., 2019)), even though inter-daily stability (IS) was impacted by the degree of cognitive impairment and reduced the potential benefit from more light exposure in these patients. Although only one influenza strain showed a significantly increased response, one other strain (IB) showed a similar trend to increase (p = 0.08).

The results from blood factors measured before and after the influenza vaccination were all in the normal range and revealed some vaccination effects (e.g., lymphocyte count) and some lower values for the older subgroup (e.g., erythrocytes and haemoglobin). There was no statistically significant difference in any blood factor between the light exposure groups, including those involved in T-cell related immune responses (e.g., the CD4 and CD8 cells and the CD4/CD8 ratio). Any differences in antibody responses were rather conveyed through the humoral and adaptive immunity driving cells, the B-lymphocytes.

**Fig. 3:**
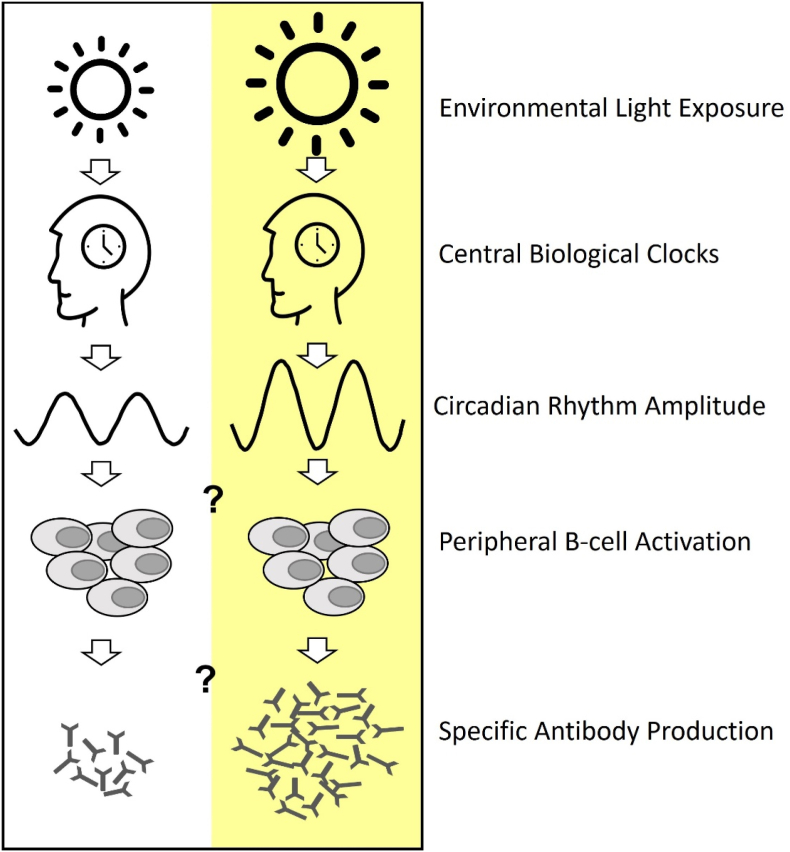
Simplified schema illustrating lower environmental light exposures (left panel) via retino-hypothalamic neuronal projections to the SCN in the brain elicit lower circadian amplitude of rest-activity cycles and probably other circadian rhythms and a consequently lower specific antibody production as it is for example known in night shift workers (Ruiz et al., 2020). The same potential pathway of action is shown on the right panel but for higher light exposures, which results in higher antibody responses. The question marks illustrate the hypothesised open research questions.

There is some evidence that more daytime light (= increased Zeitgeber strength) can lead to increased amplitude for example of melatonin secretion profiles in older institutionalised individuals (Mishima et al., 2001), or stabilised rest-activity profiles in Alzheimer patients (Van Someren et al., 1997). Therefore, it is tempting to speculate that the higher immune responses in our patient group with brighter light exposure were also indirectly facilitated by overall higher circadian rhythm stability (see also Fig. 3). However, the interconnections between the immune system, sleep, mood and circadian rhythms are rather complex (see for example a recent review (Haspel et al., 2020a)), and far from being comprehensively understood. This is also corroborated by the lack of statistically significant intercorrelations between sleep-wake and immune variables in our sample.

Other factors may have also indirectly contributed to our results, e.g., stress, emotional or other biopsychological and social aspects (bidirectionally) affect acute and long-term immune responses (Vedhara et al., 1999; Dhabhar, 2014). In our sample, we previously reported more expressions of positive emotions, greater alertness and higher quality of life over an 8 week observation period in the group with higher light exposures (Münch et al., 2017). Therefore, since these positive findings occurred in the same patient group, it may be that brighter light exposure improved immune responses not only by increasing circadian stability but also by supporting positive emotions such as improved mood, greater alertness and quality of life (Münch et al., 2017).

Lastly, we can ask why there was a significant antibody titer increase in the high light group to the H3N2 virus strain, a trend also for the IB virus strain, but not the H1N1 virus strain. For the H1N1 virus strain, pre-vaccination antibody titers were relatively low, with a large (and similar) post-vaccination ratio in both groups. The H1N1 influenza virus (‘swine flu’) was relatively new in 2012. It was declared a pandemic influenza strain by the World Health Organization between 2009 and 2010, and the vaccination against H1N1 started in 2009. Interestingly, the incidence of the disease was lowest in adults older than 65 years (Van Kerkhove et al., 2013) compared to other age-groups. Therefore, older people had may have been less infected with the H1N1 virus in previous years, resulting in lower pre-vaccination antibody loads.

Beneficial non-visual effects of brighter light exposure can obviously be provided by natural daylight (with appropriate UV-protection of skin and eyes), but also by improved electrical lighting systems (Ancoli-Israel et al., 2003; Wahnschaffe et al., 2017; Figueiro et al., 2019; Riemersma-van der Lek et al., 2008; Dowling et al., 2005; Mishima et al., 1994). In addition, it is well known that natural daylight through the skin stimulates innate and adaptive immune responses, thus preventing different diseases through Vitamin D production from Ultraviolet B (UVB) solar radiation (Hawk, 2020; Reichrath, 2020). In our dementia patient cohort, we used wrist-worn light sensors, which continuously measured illuminance. A limitation of our study may be that we could not disentangle whether participants were outside and exposed to direct sunlight (and UVB) or inside, where window glazing absorbs UVB radiation.

In summary, there may be a range of benefits from regular brighter and natural light exposures in older and frail individuals: better circadian entrainment, mood, rest-activity and eventually, also immune functions. Our preliminary findings indicate that long-term brighter light exposure in older institutionalised patients with dementia, may foster increased antibody titer production in response to the annual influenza vaccination. Such changes in antibody titer production may occur indirectly via circadian rhythm stabilisation (higher relative circadian amplitude and less inter-daily variation), leading to greater peripheral B-cell activation and antibody production, as was also hypothesised in a recent review (Palomino-Segura and Hidalgo, 2021), illustrated in a simplified model in Fig. 3. Our study, however, was not designed to dissect the highly complex underlying mechanistic pathways or to deliver antibody production kinetics.

To conclude, our findings have preliminary character. They show that brighter daily illuminance exposures, provided by mixed natural daylight and electrical lighting, might benefit older institutionalised patients with dementia in modulating specific immune responses. To disentangle possible causal relationships and their mechanistic pathways, prospective clinical studies investigating light exposure effects on these variables are warranted.

## Conflict of interest

MM, AWJ, JLS and CC are members of the Daylight Academy. CC has had the following commercial interests in the last three years (2019–2021) related to lighting: honoraria, travel, accommodation and/or meals for invited keynote lectures, conference presentations or teaching from Toshiba Materials, Velux, Firalux, Lighting Europe, Electrosuisse, Novartis, Roche, Elite, Servier, and WIR Bank.

## Funding

This study was supported by the 10.13039/100007214Velux Foundation Switzerland (Proposal 259a: Post-doctoral Fellowship), the Age Stiftung Switzerland (I-2010-015) and Sonnweid Stiftung, Switzerland.

## Declaration of competing interest

MM, AWJ, JLS and CC are members of the Daylight Academy. CC has had the following commercial interests in the last three years (2019–2021) related to lighting: honoraria, travel, accommodation and/or meals for invited keynote lectures, conference presentations or teaching from Toshiba Materials, Velux, Firalux, Lighting Europe, Electrosuisse, Novartis, Roche, Elite, Servier, and WIR Bank.

## Data Availability

Data will be made available on request.
