# Supplementary material for: Preliminary evidence that daily light exposure enhances the antibody response to influenza vaccination in patients with dementia
Source: Brain Behav Immun Health. 2022 Sep 20;26:100515. doi: 10.1016/j.bbih.2022.100515 (PMC9526132; doi:10.1016/j.bbih.2022.100515)
Supplement: Multimedia component 1 [file mmc1.docx]

**Münch M. et al.; Preliminary evidence that daily light exposure enhances the antibody response to influenza vaccination in patients with dementia**

**Supplemental Material**

**Influenza vaccination responses: effects of AGE, SEX**

Pre- and post-vaccination antibody titers did not show significant differences between the light exposure groups (p > 0.07). The interactions of LIGHT EXPOSURE GROUP with SEX and/or AGE were not significant. The H1N1 antibody titer of the pre-vaccination was significantly higher in the older (73.2 ± 12.4; n = 41) than the younger subgroup [(27.4 ± 6.2; n = 38) main effect of AGE; F_1,71_ = 8.9; p = 0.004)]. For the IB antibody titer, there were significant interactions between AGE and SEX such that pre-vaccination antibody titers were lower for older men than women (older men 35.8 ± 13.2, n = 11; older women 81.3 ± 10.4, n = 31; F_1,72_ = 6.5; p = 0.01). IB antibody titer ratios were significantly higher for older men only in the high light exposure group (26.3 ± 10.6; n = 5) than women of the same group (mean 6.2 ± 2.9 SD; n = 17; interaction LIGHT EXPOSURE GROUP x SEX x AGE; 5.6 ± 1.8; F_1,72_ = 9.7; p = 0.003).

**Supplemental Table S1:** Summary of excluded patients

| **Participant excluded (#)** | **Reason** |
| --- | --- |
| 49 | Retraction of consent |
| 66, 76 | Deceased |
| 81 | Transferred to psychiatry (> 3 weeks) during an acute episode |
| 73, 82, 99 | Eye disease (Glaucoma, Retinitis Pigmentosa) |
| 8, 40, 70 | No activity watch data |
| 6, 29, 38, 52, 84 | Insufficient light data |
| 3, (8) 21, 39, (40), (52), 54, 55, 59, 91 | Not 2 blood samples and/or no influence vaccination (in brackets = patients already excluded from analysis) |
| 7, 44, | Leucocyte cell count > 15’000 / µl (likely due to acute infection) |

**Supplemental Table S2:**

| \| **Strain** \| **Groups** \| **GMT** \| **Seroprotection Rate** \| **Seroconversion Rate** \|  \| \| --- \| --- \| --- \| --- \| --- \| --- \| \| **H3N2** \| **Pre: low** \| 116.51 \| 92 \| - \|  \| \| **(n=78)** \| **high** \| 88.82 \| 80 \| - \|  \| \|  \| **Post: low** \| 272.15 \| 100 \| 34 \|  \| \|  \| **high** \| 309.88 \| 95 \| 38 \|  \| \| **H1N1** \| **Pre: low** \| 19.97 \| 21 \| - \|  \| \| **(n=79)** \| **high** \| 25.11 \| 43 \| - \|  \| \|  \| **Post: low** \| 218.35 \| 85 \| 74 \|  \| \|  \| **high** \| 171.51 \| 83 \| 73 \|  \| \| **IB** \| **Pre: low** \| 40.10 \| 58 \| - \|  \| \| **(n=80)** \| **high** \| 32.67 \| 45 \| - \|  \| \|  \| **Post: low** \| 112.26 \| 75 \| 38 \|  \| \|  \| **high** \| 128.41 \| 90 \| 43 \|  \| |
| --- | --- | --- | --- | --- | --- | --- | --- | --- | --- | --- | --- | --- | --- | --- | --- | --- | --- | --- | --- | --- | --- | --- | --- | --- | --- | --- | --- | --- | --- | --- | --- | --- | --- | --- | --- | --- | --- | --- | --- | --- | --- | --- | --- | --- | --- | --- | --- | --- | --- | --- | --- | --- | --- | --- | --- | --- | --- | --- | --- | --- | --- | --- | --- | --- | --- | --- | --- | --- | --- | --- | --- | --- | --- | --- | --- | --- | --- | --- |
| **Supplemental Table S2:** Shows the geometric mean antibody titers (GMT) for each influenza strain before and after vaccination and for both sub-patient groups (low light, high light group respectively). The sero-protection rate indicates the portion of the cohort with a post-vaccination titer > or 40 (in %). The seroconversion rate shows the proportion of the cohort with 4-fold GMTs (in %). |
